# Integrating musealized archaeological sediment collections into current geoarchaeological analytical frameworks for sustainable research practices

**DOI:** 10.1016/j.mex.2024.102897

**Published:** 2024-08-09

**Authors:** Stefano Costanzo, Marta Pappalardo, Elisabetta Starnini, Elena Rossoni-Notter, Olivier Notter, Abdelkader Moussous, Miguel Soares-Remiseiro, Paola Fermo, Mauro Cremaschi, Andrea Zerboni

**Affiliations:** a Dipartimento di Scienze della Terra “Ardito Desio”, Università degli Studi di Milano, Milan, Italy; b Dipartimento di Scienze della Terra, Università di Pisa, Pisa, Italy; c Dipartimento di Civiltà e Forme del Sapere, Università di Pisa, Pisa, Italy; d Museum of Prehistoric Anthropology of Monaco, Monaco; e The interdisciplinary Center for Archaeology and Evolution of Human Behaviour, Universidade do Algarve, Faro, Portugal; f Dipartimento di Chimica, Università degli Studi di Milano, Milan, Italy

**Keywords:** Archaeology, Geoarchaeology, Thin section micromorphology, Biomarkers, SedaDNA, Human ecology, Museum collections, Geoarchaeological frameworks for Human Ecology studies

## Abstract

We present a review of the latest framework achievements in geoarchaeological sciences applied to microstratigraphic and biomolecular studies of prehistoric archaeological contexts, highlighting the importance of musealized archaeological stratigraphies. We assess how today's scientific and technological accomplishments can be tailored for archaeological human ecology studies with analytical ensembles that provide unprecedented results. Sampling and processing workflows originating from resin consolidation and thin section micromorphology of undisturbed blocks of archaeological soils and sediments, guarantee subsampling accuracy at the micrometre scale granting access to individual components otherwise impossible to target: the achievable information yield makes even the smallest soil samples potential sources of pioneering discoveries. Yet, archaeological excavations are still the primary mode of retrieving new soil samples. We argue that, when dealing with archaeological sites that were excavated and partially musealised in the past, the exploration of related museum collections should be prioritized as perspective source of new study samples. Analysing old and potentially very informative samples, with an approach that we define as “Green Archaeology”, may represent a source of well-structured primary data as well as a means for planning new excavations, delivering novel discoveries while safeguarding site integrity and promoting Third Mission valorisation of sites and heritage dormant collections.

## Review Highlights

• Geosciences and Life Sciences applied to archaeological contexts are at an all-time high.
• Museum collections, dormant scientific documentation and exposed site stratigraphies are key for site valorisation and sustainable practices.

## Graphical abstract

**Figure:**
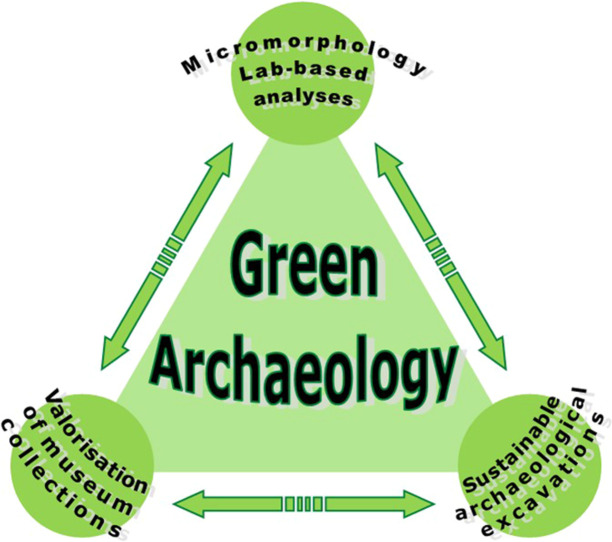

Specifications table

**Table:** 

Subject area:	Environmental Sciences
More specific subject area:	Geoarchaeology
Name of the reviewed methodology:	Geoarchaeological frameworks for Human Ecology studies
Keywords:	Archaeology; Geoarchaeology; Thin section micromorphology; Biomarkers; sedaDNA; Human ecology; Museum collections
Resource availability:	Datasets are contingent to the cited papers’ proprietary resources
Review question:	What is the state of the art in the study of archaeological contexts? Can framework achievements be implemented to ensure best practice towards sustainability?

## Background

Past adaptations and survival strategies of our ancestors and other extinct hominin species left traces in the stratigraphic record of the Quaternary, reaching from landscape-scale modifications down to molecular interactions embedded into sediments and soils [[1], [2], [3], [4]]. During the last few years, engineering progresses have been conditioning an ever-growing capability of extracting biochemical, geochemical and isotopic data with exceptional sensibility to low and trace concentrations, prompting archaeological scientists and geoscientists to incorporate such advancements into their own research [[5], [6], [7], [8], [9], [10], [11]]. First-hand delving into the exploration of the molecular sedimentary record highlighted its value as proxy data for paleoenvironmental reconstructions framed within precise and improved chronological contexts. Beside huge developments in geochronology and paleoenvironmental reconstruction, the exploration of the molecular record represented a milestone achievement thanks to the possibility of detecting trace amounts of DNA and species-specific/land use-specific organic compounds (biomarkers) [[12], [13], [14], [15]]. These have been found to be subjected to molecular binding within the crystalline structures of the clastic record of archaeological stratigraphies, and a rapid surge of new discoveries upon the lineages of hominin species, as well as their ecology and relationship with local environments, contributed to parallel improvements of the technical knowledge for extraction methods [[16], [17], [18], [19], [20], [21]]. What was until recently hindered by an incomplete comprehension of the preservation of organic compounds, and by and inadequate instrumental detection limit, is nowadays accessible by application of rigorous yet achievable interdisciplinary analytical frameworks.

Nonetheless, many such new efforts still rely on freshly collected sedimentary material gathered from ongoing archaeological excavations – a practice that is, by definition, destructive, and non-replicable [22]. Here we propose a substantial spin on the execution of new analyses, prompting researchers to consider investigating the existence and accessibility of old in-situ open trenches and stratigraphy remnants, but also, most importantly, the availability of archaeological sediment/soil samples collected during excavations since the 1800s today belonging to museum heritage collections. This research strategy, which we define as “Green Archaeology”, pushes for a thorough valorisation of the available raw archaeological data, with the aim of fine-tuning and minimising new excavations, thus safeguarding site integrity and guaranteeing sustainable practices.

### Method details

Recent discoveries in the field of archaeology and evolutionary anthropology owe their impact to the advances in understanding archaeological sediment/soil's microscopic properties and the preservation of biomolecules in the sedimentary record [17,[23], [24], [25], [26], [27], [28]], supported by engineering of instrumentation ever-lowering the detection limit of trace amounts of organic compounds extracted from soil samples. Joint efforts of archaeologists, geoscientists, and life scientists employing microscopy techniques on soil samples and archaeological sediments to pilot laboratory-based microsampling for biomolecular detection, have been pushing boundaries towards a multifaceted comprehension of the depositional and ecological history of archaeological contexts [12,17,26]. Such efforts found proof of deliverability and practicality in recent publications upon prehistoric cave dwelling, *Homo* lineages and interspecies space-sharing [6,17]. Simultaneously, new standards of application and good practice were set in terms of interdisciplinarity and rigor of workflow, ultimately inducing a leap forward among working groups that, in recent years, have been expanding their lab equipment and providing training and mentorship at all levels.

Yet, in the strive for new ground-breaking discoveries, the very execution of avant-garde interdisciplinary analytical frameworks still relies on the exploitation of archaeological sites by means of stratigraphic excavation, implying an irreversible consumption of pristine deposits. Nonetheless, the possibility of reappraising old and well-preserved trench sections, and studying musealised samples of archaeological stratigraphy, is an investigative route that is oftentimes overlooked despite holding remarkable potential.

Many of today's most famous prehistoric sites have been known and excavated for decades. Among these, cave sites have been at the core of paleoanthropological discoveries thanks to their intrinsic qualities: caves and rock shelters are privileged hubs of domestic and practical aggregation for humans and other species, with deep stratigraphies enclosing dense records. Moreover, the sheltered settings guarantee protection of the primary record, which is generally less subjected to erosion compared to open-air sites, thus bearing better-preserved osteological record, accretional stratigraphy, material culture and primary biochemical properties. Additionally, geogenic processes such as cave clast deposition, functioning as trap for wind-blown sediments, and speleothem accretion, make cave archaeological contexts more reliable for complex dating and the study of hinterland climatic and ecological variations. However, cave stratigraphies bear the downside of being spatially constrained to the very cave's own size, oftentimes adding up to but a few tens of cubic meters. Across the 1800′s and first half on the 1900′s, limited or non-existent Science, Technology, Engineering and Mathematics (STEM) accomplishments were in some cases compensated for by archaeologists who dedicated great care and artistry directed towards the representation and characterization of archaeological stratigraphies [[29], [30], [31], [32]]. However, the main *corpus* of anthropological and paleontological data came from the recovery and purely typological study of bones, burials, and material culture (i.e. lithics, ceramics, metal, semiprecious elaborates, and occasional decay-prone objects such as wooden tools, basketry, leather and textiles). The sought for “missing links” and a tendency to a *Wunderkammer* approach inevitably caused a fast-paced consumption of the already limited stratigraphic record [[33], [34], [35]], today translating into doubts concerning the reliability of early authors’ claims about chronologies, stratigraphic correlations and paleoanthropological interpretations. Whereas accessibility to collections of objects is granted for new studies and appraisals, lost sediment cannot be retrieved, and present-day archaeological endeavours in long-known cave sites are relegated to discontinuous spare portions of stratigraphy that may be less rich in terms of stratigraphic significance and biochemical information yield.

Nevertheless, exploration of scientific documentation may reveal that *sedimentological specimens* collected during old excavations were kept along with artefacts in institutional storages (museums, universities, administrative authorities) for perspective later studies. Such *specimens*, or samples, can be easily included within up-to-date analytical frameworks upon evaluation of the accuracy of their recording data.

**Fig. 1:**
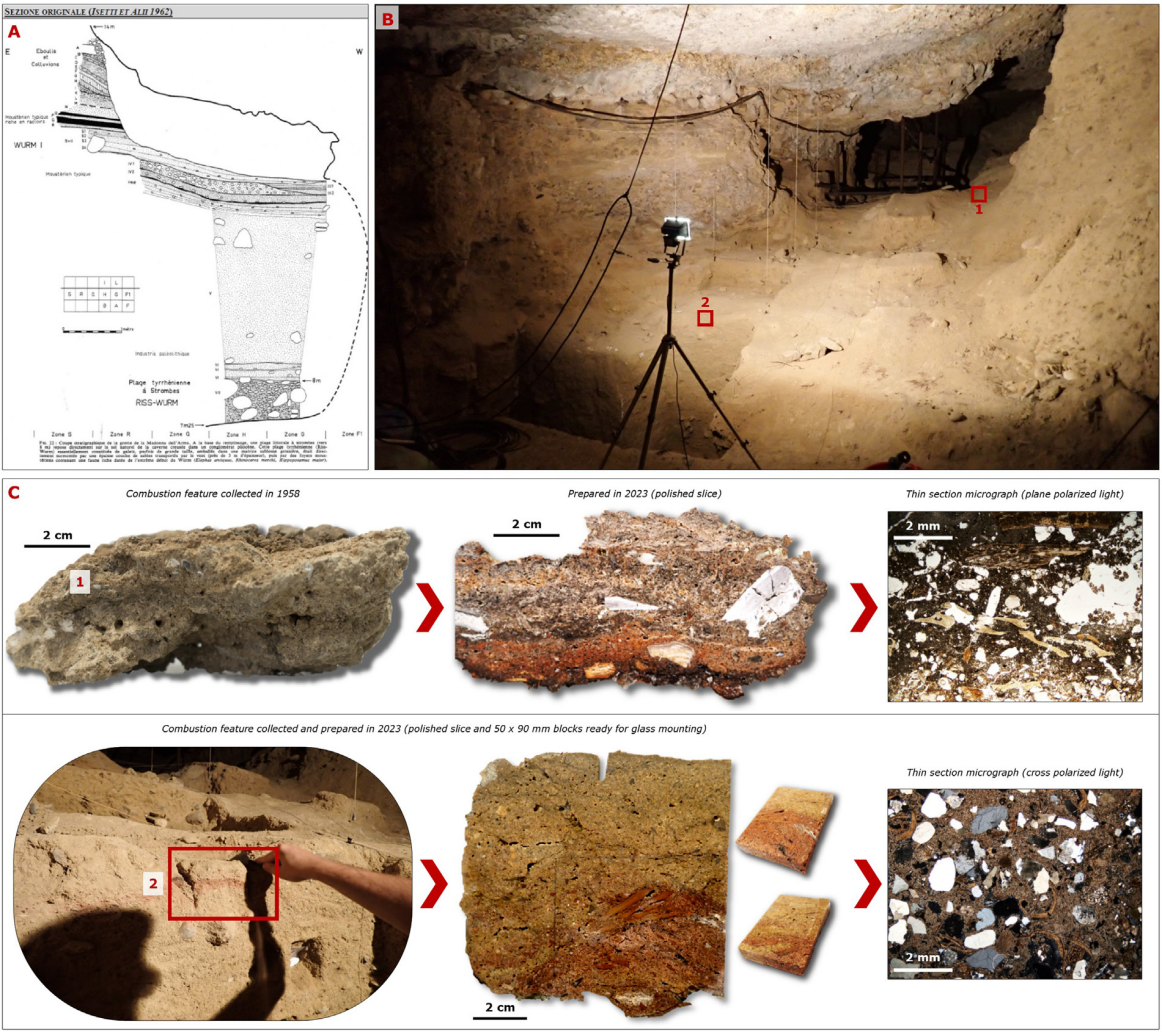
Investigations at Madonna dell'Arma cave, Sanremo, Italy. A) The original section drawing from the 1958–1962 excavations, encompassing investigated stratigraphy reaching the bottom “Tyrrhenian” (MIS 5e) beach deposits. Evidence for Neanderthal dwelling was found throughout the sequence in the form of abundant lithic industry. B) The cave as it appears today. In the background, reinforced with iron pipes, the deep trench from 1958 to 1962 excavations. In the foreground, a more recent trench revisited and sampled in 2023. The red boxes indicate the position of samples shown in panel (C). C) Hearth fragments recorded and sampled between 1958 and 1962 (1) and 2023 (2), shown before and after resin impregnation and slicing. 1958–1962 samples were retrieved in 2023 from the storage of the Museo Civico di Sanremo. The trenches excavated sixty years ago are still well-preserved and exposed, and new samplings from one of the exposed sections were conducted in 2023 following a reassessment of the 1962′s stratigraphic interpretations [48]. Old and new samples were consolidated in resin and are undergoing pilot micromorphological analyses.

**Fig. 2:**
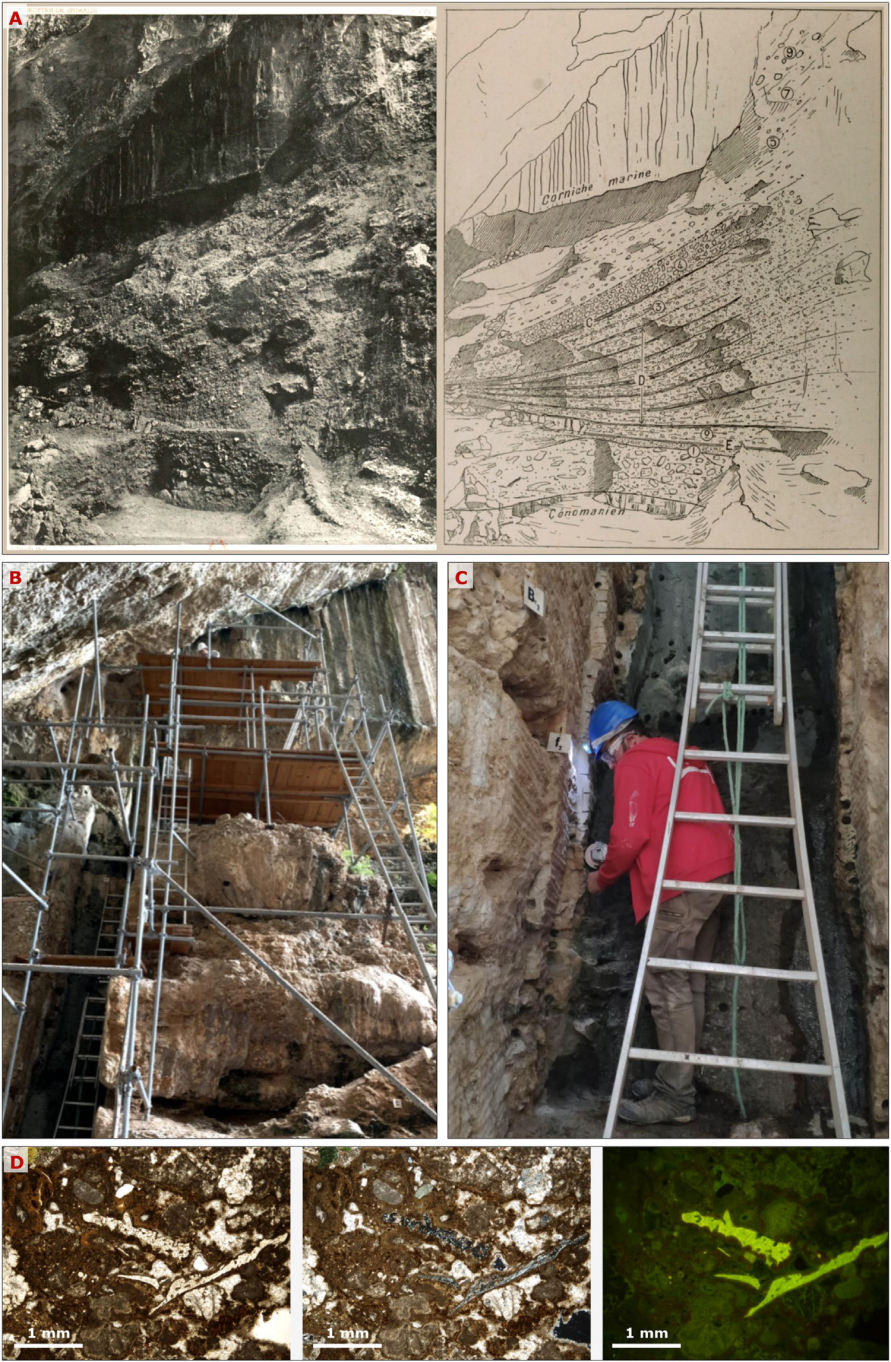
Investigation at the Prince of Monaco's Cave at Balzi Rossi, Italy. A) Original photograph and section drawing from the early 1900′s (Museum of Prehistoric Anthropology of Monaco scientific documentation). Excavations were carried out throughout the 19th and 20th centuries and investigated most of the archaeological and geological stratigraphy. B) Newly constructed infrastructure providing access to the preserved trench walls. C) Sampling of the lithified archaeological deposit. D) Thin section micromorphology of preserved layers (left to right: micrographs of a concentration of bone fragments in plane-polarised light, cross-polarised light, and autofluorescence) unveils details on site formation and domestic practices invisible during excavation. Resin consolidated blocks are archived and available for further analyses.

The feasibility of this approach was demonstrated during recent activities by the interdisciplinary SPHeritage Project [Anon., 36]. The project aimed to investigate behavioural and land use responses of Palaeolithic communities to Quaternary climatic variability, sea-level fluctuations, and related environmental changes in the *Balzi Rossi* archaeological area and nearby region of the Liguro-Provençal coastline (NW Italy – SE France), where numerous coastal caves and rock shelters were occupied since the arrival of *Homo neanderthalensis* and even earlier hominin species [[37], [38], [39]]. Therein, nearly 180 years of archaeological excavations at many of the caves and rock shelters of the area substantially reduced the possibility to identify pristine stratigraphic records [[40], [41]]. Nonetheless, several actions were performed for the research purpose: residual strips of archaeological sediments were identified as targets for updated analyses and radiometric dating, and most crucially the documentation phases of the project and the exploration of paper archives and collections accumulated from decades of excavations, unveiled that the *Musée d'anthropologie préhistorique de Monaco* (Principality of Monaco) and the *Museo Civico di Sanremo* (Italy) hosted several sediment specimens labelled with their exact stratigraphic position by earlier excavation teams (XYZ location, Stratigraphic Unit number, brief description). The specimens come from the *Balzi Rossi* and other caves from the surrounding Italian, Monegasque and French rivieras, and comprise sublithified fragments of fire-related features, beachrock-like concretions, ashy-lime concretions containing bone fragments, shell fragments, charcoal, and other ecofacts that have already undergone renovated studies [[41], [42]]. A remarkable discovery are some brittle combustion feature fragments collected in 1958–1962 from another small cave belonging to the regional complex, the *Madonna dell'Arma* Cave (Sanremo, NW Italy), associated with Neanderthal dwelling [[43], [44]] (Fig.1). The retrieved combustion feature fragments, 15 in total from four different stratigraphic units, were 3D recorded to create virtual copies for the archive of the museum and web dissemination, consolidated with resin [45], and then directed to analyses. A recent visit to the cave disclosed the presence, on one preserved trench section from 1962, of another combustion feature still in situ. This was sampled and consolidated as well, and together with the heritage 1958–1962 samples is now being analysed in an effort to valorise the heritage collections and extract novel data with state-of-the-art science prior to any further excavation. Moreover, caves from the region in most cases still retain tall portions of preserved stratigraphy and hanging chunks of encrusted and lithified archaeological sediments, such is the case of the *Cave of the Prince of Monaco* [37,[46], [47]]. These are difficult to investigate with further excavations due to logistic risk posed by their steepness and small surface/height ratio of the deposits, but whenever reappraisal is needed for assessing dubious formation processes and radiometric chronologies, old drawings serve as guides for wall sampling aided with ladder systems (Fig. 2).

**Fig. 3:**
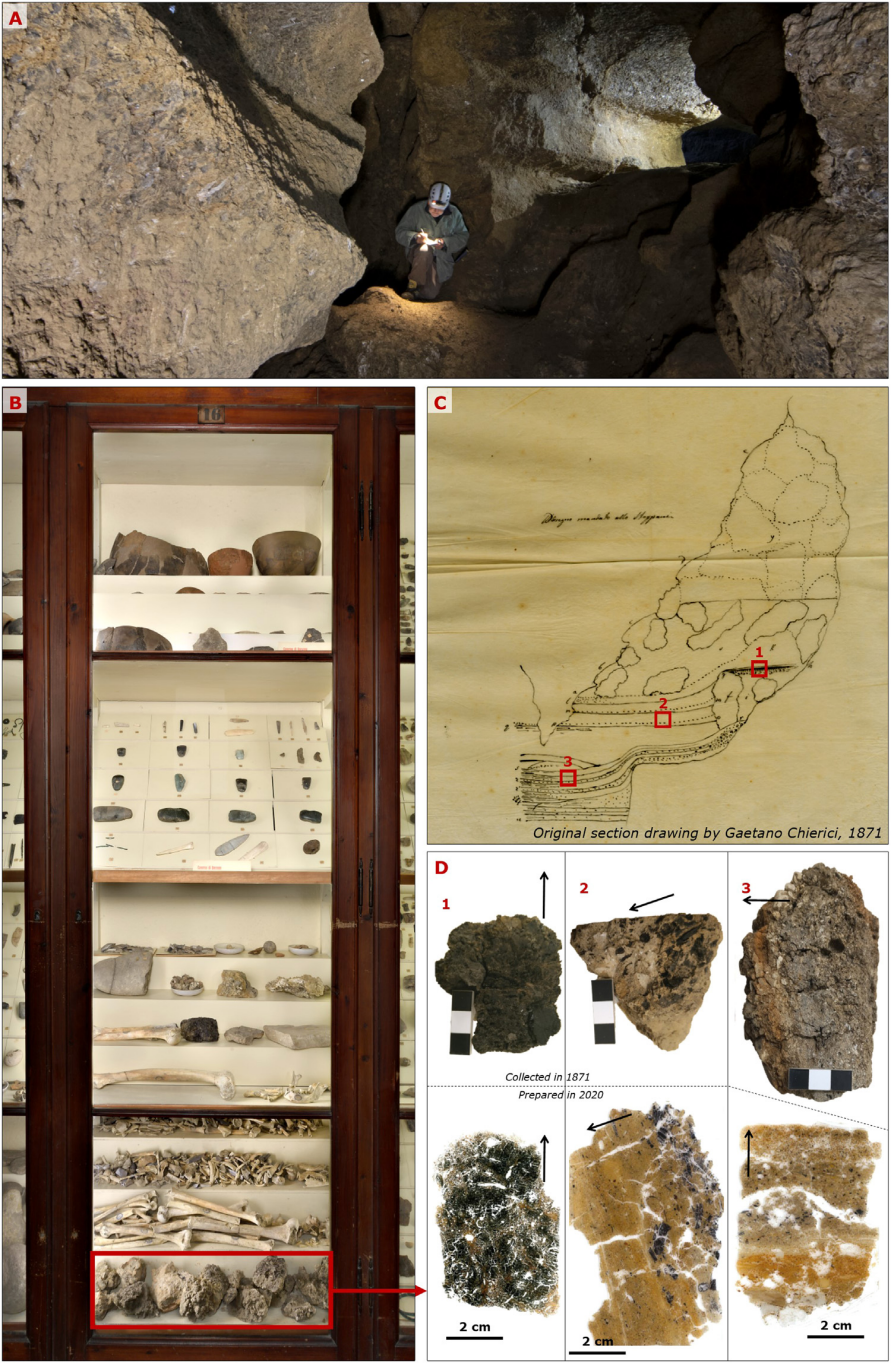
Heritage sediment samples from the Tana della Mussina cave, Italy. A) Photograph of the interior of the cave as it appears today. The pristine cave deposit used to reach halfway up the height of the chamber's vault. B) Stratigraphy specimens were collected by Gaetano Chierici during excavations in the 1870′s and are today displayed at the Museo Chierici di Paletnologia (Reggio nell'Emilia, Italy). C) Original section drawing produced by Gaetano Chierici in 1871. Today the cave's archaeological stratigraphy is exhausted, and the red boxes reported on the drawing indicate the samples displayed in the museum, illustrated in panel D. D) Stratigraphy blocks as they were gathered from the museum display (above), and how they appear as glass-mounted thin sections.s Images adapted from [51].

Another case of successful application of interdisciplinary reappraisal of samples collected in the past comes from the study ensemble carried out on the *Tana della Mussina* (Albinea – Reggio nell'Emilia), a cave site from a gypsum karst locality of the northern Apennines mountains of Italy attended in the Eneolithic and Bronze age. Therein, archaeological excavations of the outer chambers were conducted in 1871 by the Italian paletnologist Gateano Chierici [[49], [50]], followed in recent times by an intervention directed at investigating the cave's interior and the speleothem record [51]. Notably, Chierici himself collected soil fragments that are currently displayed at the *Musei Civici di Reggio Emilia* together with finds and historical drawings. Such fragments were recently subsampled to carry out geoarchaeological reassessments and thin section micromorphology [[51], [52]], which provided support in clarifying certain aspects of site formation processes related to the interplay between human cave dwelling and karst water circulation (Fig. 3). Additionally, resin consolidation aided in microsampling of single-piece charcoal fragments for radiocarbon dating of layers that are no longer present in the cave's primary setting.

Efforts on the study of musealised sediment specimens have been conducted for open-air sites as well. A 60×50 cm slab of archaeological stratigraphy collected in 1887 by geologist and archaeologist Giuseppe Scarabelli [53] in the site of Monte Castellaccio (Imola, NW Italy) – a site belonging to the Bronze Age Terramare Culture of the Po Plain of N Italy [54] was studied in 1996 [55] as part of a valorisation programme promoted by the local museum of Imola. The slab was reassessed following the original drawings and sampled for micromorphology in order to elucidate site formation processes that the original authors could not identify.

These case-studies of beneficial use of trenches and samples dug and collected in the past, provide snippets of a most comprehensive and well-structured interdisciplinary workflow that was graphically summarized in a recent article [6]. Therein, the authors highlight the importance of a carrying out soil micromorphology applied to archaeological sediments [[56], [57], [58]] prior to other techniques, pointing out that the interpretation of microscopic features provides a guide for subsequent interventions from each branch of geology, chemistry, palaeontology, geochronology and molecular biology that can weigh in to provide answers to complex and unorthodox archaeological and paleoanthropological research questions. Namely, [7] proposes a baseline approach that begins with the collection, from the excavation site – or from museum collections, as we are remarking here –, of undisturbed and oriented blocks of sediments that are representative of stratigraphic features deemed worthy of sampling due to visible or foreseeable properties to be tested and measured in a laboratory setting. Blocks can be any size depending on sampling permits, viability, and structural and cohesive properties of the sediment, ranging from small cubes measuring a few centimetres per side, up to pluridecimetric standing columns or flat beds possibly collected with the aid of metal/plastic/plaster casts and wooden boards. Blocks ought to be recorded in their 3D position within the stratigraphy, to allow retaining their spatial orientation while being subjected to further analyses far from the collection site. Studying undisturbed blocks in a laboratory setting allows manifold approaches that have low chances of success when carried out in the field, especially those requiring the implementation of sterile protocols. Lab conditions ensure still ambient air, clean clothing, comfortable use of gloves, masks and goggles, better lighting, and a generally more agreeable environment for carrying out delicate subsampling. Moreover, resin impregnation of the blocks allows for long-term storage of the hardened sediment samples in their pristine spatial architecture [59], and it has been proven that such procedure does not alter biomolecular characteristics regardless of the storage time and employed resin [6,27]. Resin-hardening of sediment blocks using low-viscosity, isotropic, and optically transparent polymers (e.g., polyester resin diluted with styrene in a 7:3 ratio) is also the first step for producing thin sections destined to micromorphological analysis [45]. Micromorphology in archaeology adopts standardized terminologies to describe microscopic evidence, allowing mutual comprehension between researchers. Such terminologies are derived from the description [60] and interpretation [61] of microscopic pedofeatures and evolution of extant and ancient soils (e.g., paleosoils; [[62], [63]], and are efficiently applied to archaeological soils and sediments [[56], [57], [58]]. Following these principles, the observation and interpretation of microscopic textural, structural, organic and pedogenetic features was proven to be a compulsory operation for ensuring spatial validity of microbulk subsampling [6]. In the field, it is intrinsically impossible to perform microbulk samplings that ensure correct sediment targeting while avoiding, e.g., faunal/plant disturbances (bioturbation) or sediment mixing (pedoturbation) that may have caused deep blending of strata while incorporating allochthonous DNA or biomarkers into old sediments. Conversely, a thin section represents the mirror image of the hardened block's surface, which can be drilled for microsampling at the micrometre scale avoiding disturbances by using the thin section itself as guide. High-precision microsamplings can thus be destined to DNA analyses (both from the sedimentary matrix and single fragments of bones/coprolites) and other techniques (Table 1) such as, but not limited to, lipid and wax analyses by Gas Chromatography - Mass Spectrometry (GC–MS), nanopaleontological analyses, Fourier Transform infrared spectroscopy (FTIR), X-ray diffraction (XRD), X-ray fluorescence (XRF), and radiometric dating. Thin sections and hardened blocks, owing to their flat polished surfaces, are also ideal for executing spatially resolved non-invasive or micro-invasive chemical and elemental characterizations and mappings, such as scanning electron microscopy with energy dispersive spectrometry (SEM-EDS), laser ablation multicollector mass spectrometry (LA-ICP-MS) and spatially resolved dosimetry.

**Table 1:** List of techniques and the general types of data obtainable.

Technique	Type of data obtained
Thin section micromorphology	Identification of microscopic sedimentary structures and geogenic/pedogenic/biogenic features and components
Scanning electron microscopy with energy dispersive spectrometry (SEM-EDS)	Spatially resolved quantitative detection of elements (light and heavy)
Gas chromatography – Mass spectrometry (GC–MS)	Separation and detection of organic compounds such as lipids, waxes and resins
Laser ablation multicollector mass spectrometry (LA-ICP-MS)	Quantitative detection of trace elements in solid materials
Fourier Transform infrared spectroscopy (FTIR)	Detection of organic and inorganic molecules
X-ray diffraction (XRD)	Quantitative detection of crystalline substances
X-ray fluorescence (XRF)	Quantitative detection of heavy elements (*Z* > 12–13)
(Nano)paleontological analyses	Identification of index fossils

In essence, the collection, consolidation and archiving of undisturbed blocks of sediment unlocks an array of analytical possibilities of non-negligible relevance, and the action should be internalized as a baseline practice of any archaeological excavation team regardless of the subfield expertise of each team member. This is especially crucial when archaeological excavations take place in areas of the world with difficult and expensive logistics – meaning fieldwork seasons may be separated by several years at times –, or when national legislations impose integral backfilling of excavation trenches, which again may be revisited only years later despite more accessible logistics. However, in the case of imposed backfilling, laws and good practice demand the positioning of non-woven fabric (or similar textiles) at the base and along the sides of the excavated trenches to define the reached limits. A quick removal of the backfill creates access to the extant trench sections, making new minimally-invasive samplings easy to plan and perform.

**Fig. 4:**
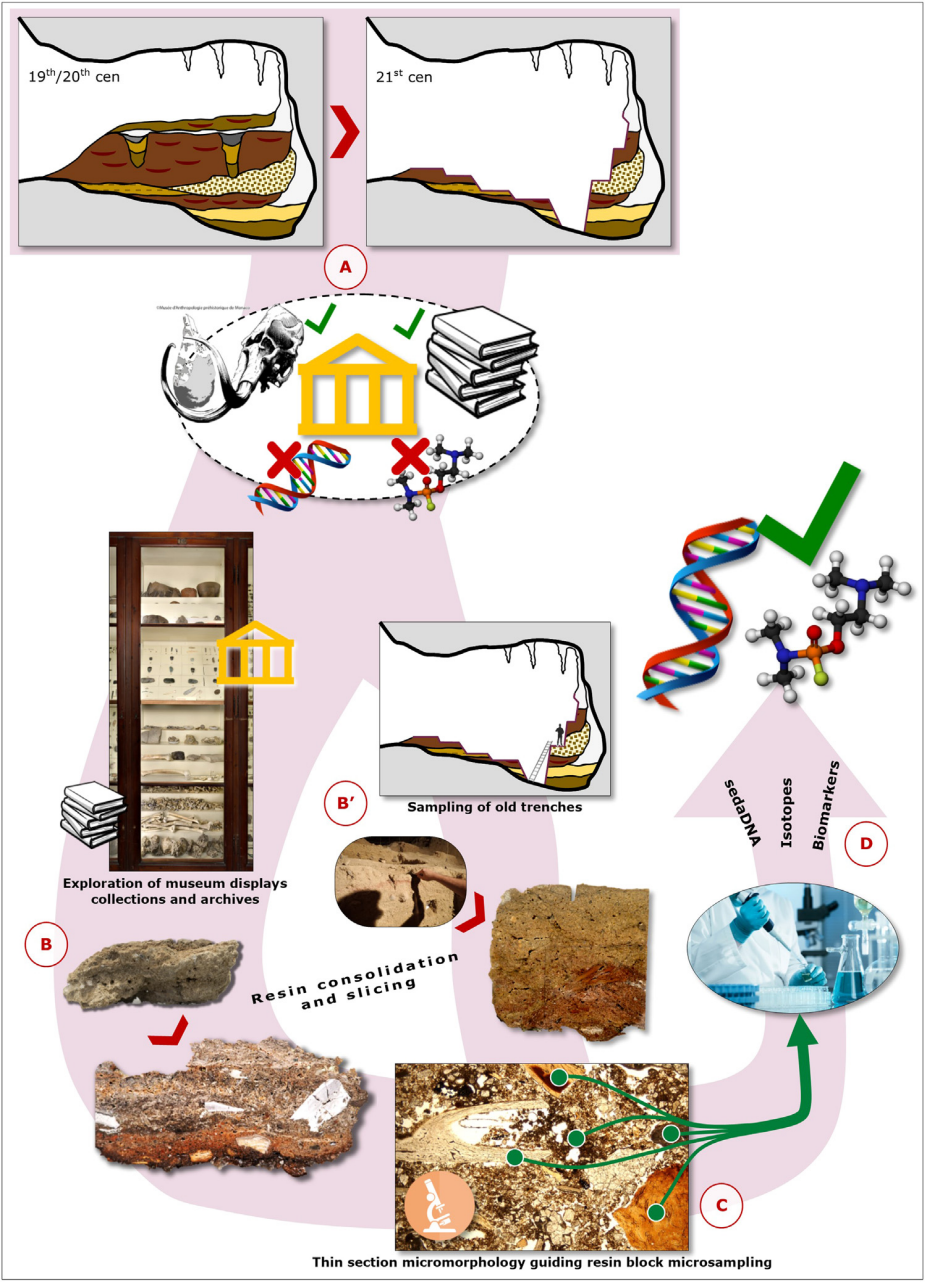
Conceptual workflow. Assuming that an archaeological cave or open-air site presents today only a fraction of unexcavated stratigraphy, we recommend undertaking workflow strategies based on reappraisal of underexploited trench sections and musealized soil and stratigraphy samples through micromorphology and subsequent analyses, instead of planning new excavations unless strictly necessary. A) Archeological excavations carried out in the past enriched our collective scientific knowledge, albeit consuming site stratigraphies irreversibly. Advanced STEM studies often simply didn't exist, leaving a gap in the potential information yield. B-B’) In order to fill the gap, samples directed to STEM studies through micromorphology may be retrieved from museum collections and/or from open trenches produced during previous excavations that were not subjected to integral back-filling. C) Thin section micromorphology, on top of the information yield produced by the technique in its own respect, provides a guide for directing direct analysis on the thin slice or microsampling of the specular resin consolidated blocks. D) The microsamples gathered during the previous steps can be directed to several analytical lines to identify proxies such as, but not limited to, sedaDNA, isotopic fractionation, and biomarkers.

This approach, translated into a framework of valorisation and sustainability of archaeological practices, is likely to be repeatable for any site with long-term curated archives, representing an all-round advantageous workflow (Fig. 4): old specimens from excavated portions of archaeological deposits, or new samples retrieved from the walls of formerly excavated trenches, may yield remarkable information density, acting both as primary source of novel data and as a guide for subsequent on-site interventions to be calibrated accordingly. This promotes a more accurate stratigraphic positioning of evidence, and the substantial aim of safeguarding site integrity – as opposed to executing new and potentially harmful excavations for the pursue of fresh samples. In the future, our “Green Archaeology” concept can be taken as a conceptual approach heading towards a more sustainable geo-archaeological practice regardless of the perspective studies and techniques to be performed, both from an economic point of view and in terms of saving archaeological deposits that must be considered, indeed, a non-renewable resource.

## Conclusion

STEM techniques applied to research focused on archaeological soils and sediments are at an all-time high, and working groups are catching up accordingly. However, primary sourcing of new study samples still heavily relies on archaeological excavations, a practice that guarantees documental reliability yet implies consumption of pristine stratigraphies, ambient disruptions and enhanced soil loss. To overcome these downsides, we suggest a new and sustainable approach that we define as “Green Archaeology”, a research approach that advocates for the exploration of documental archives and storages for retrieving heritage collections of soil samples and sediment specimens to be analysed with cutting-edge technologies, with the aim of postponing and fine-tuning further excavations whilst valorising material that may be conveyor of significant discoveries. Furthermore, it is firmly suggested to incorporate the collection, consolidation and archiving of undisturbed soil samples as a baseline practice for all archaeological excavations, to guarantee future executability of lab-based analytical workflows.

## Ethics statements

This work does not contain data collected from social media platform nor involved gathering demographics data. Permission has been obtained for use of copyrighted photographic material.

## CRediT authorship contribution statement

**Stefano Costanzo:** Conceptualization, Visualization, Writing – original draft. **Marta Pappalardo:** Writing – review & editing, Project administration, Funding acquisition. **Elisabetta Starnini:** Writing – review & editing. **Elena Rossoni-Notter:** Writing – review & editing. **Olivier Notter:** Writing – review & editing. **Abdelkader Moussous:** Writing – review & editing. **Miguel Soares-Remiseiro:** Resources, Writing – review & editing. **Paola Fermo:** . **Mauro Cremaschi:** Writing – review & editing. **Andrea Zerboni:** Conceptualization, Writing – original draft, Project administration, Funding acquisition, Supervision.

## Declaration of competing interest

The authors declare that they have no known competing financial interests or personal relationships that could have appeared to influence the work reported in this paper.

## Data Availability

No data was used for the research described in the article.
